# FEASIBILITY OF INTEGRATED TECHNOLOGIES TO PROMOTE LEISURE ACTIVITY ENGAGEMENT AMONG ASSISTED LIVING RESIDENTS

**DOI:** 10.1093/geroni/igac059.3019

**Published:** 2022-12-20

**Authors:** Phyllis Gaspar, Elena Do, Junhyoung Kim, Angela Chow, Adam Sobol, Raia Francisco, Lisa McFarland

**Affiliations:** CareBand Inc., Sioux Falls, South Dakota, United States; CareBand Inc., Chicago, Illinois, United States; Indiana University, Bloomington, Indiana, United States; Indiana University Bloomington, Bloomington, Indiana, United States; Careband Inc., Chicago, Illinois, United States; CareBand Inc., Chicago, Illinois, United States; Axiatp, Zionsville, Indiana, United States

## Abstract

Research suggests that advanced technologies, such as wearable technology and tablets, can serve an important role in the cost-effective, accessible delivery of health promotion that is individualized, immersive, and engaging for users. The present study was designed to test the feasibility and acceptability of two integrated technologies among assisted living facility residents: CareBand’s wearable technology and Eperture’s RememberStuff (R/S) tablet platform. The purpose of the qualitative component of the project was to provide preliminary information from the perspective of the assisted living facility participants on these two technologies. Another goal was to assess the challenges of their participation. Drawing on qualitative thematic analyses of data collected from semi-structured interviews with 16 participants (four staff members and 12 residents) prior to and following the 6-week integrated technology program, three themes emerged as positive outcomes of the technology experiences: (a) leisure activity engagement, (b) exploration of tablet features, and (c) cognitive stimulation. This finding suggests that these technologies may enhance leisure engagement and cognitive function. On the other hand, there were three challenges that participants experienced while participating in the program: (a) inability to grasp functionality, (b) need for continued education for the technology, and (c) negative stereotypes toward technology use. These challenges provide guidance for future protocol design in follow-up studies to further explore the integrated technology efficacy for older adult users. The practical implications of this study and suggestions for health professionals are discussed.

